# Supplementary material for: Antioxidant-Mediated Modification of Citral and Its Control Effect on Mildewy Bamboo
Source: Polymers (Basel). 2022 Nov 1;14(21):4652. doi: 10.3390/polym14214652 (PMC9657708; doi:10.3390/polym14214652)
Supplement: Supplementary file 1 [file polymers-14-04652-s001.zip › polymers-1948883-supplementary.pdf]

Supplementary information

Preparation of Antioxidant-Modified Citral

Briefly, The appropriate amount of citric acid was mixed with the propylene glycol solution (pH: 6.0) [29] and stirred in a heat-collecting magnetic stirrer until the citric acid dissolved. 2 mL (10% of the total volume of the solution) of anhydrous ethanol and 2 mL (10% of the total volume of the solution) of deionized water were added to the test tube with a pipette, followed by the addition of 3.5 g of citral and 0.035 g of natural antioxidant [30]. The solution to be measured was made up to 20 mL with the aforemen-tioned propylene glycol solution. The appropriate amount of propylene glycol with a pH of 6.0 were added. The solutions to be tested were stirred with a magnetic stirrer at 100 rpm for 0.5 h, and, finally, the solutions to be tested were prepared with TP-C, ASC-C, and TF-C at 175 mg/mL and natural antioxidants at 1.0%. Similarly, the antioxidant-modified citral was prepared as described above at a 150 mg/mL con-centration with the addition of 0.8% natural antioxidants and at a 125 mg/mL concen-tration with the addition of 0.6% natural antioxidants.

Antioxidant-Modified Citral DPPH Radical-Scavenging Rate

Briefly, 1,1-Diphenyl-2-trinitrophenylhydrazyl radical 2,2-diphenyl-1-(2,4,6-trinitrophenyl) hydrazyl (DPPH) solution was prepared [31]. Then, 0.01 g DPPH was dissolved with the appropriate amount of anhydrous ethanol. The solution was poured into a 100 mL volumetric flask and shaken well. Then, the solution was poured into a 100 mL volu-metric flask and its volume was fixed by adding anhydrous ethanol solution and shak-ing the mixture. DPPH solution (20 mL) was added to a 100 mL volumetric flask, diluted 5 times with anhydrous ethanol solution, and placed at 4 °C.

29. Shi, C.; Zhao, X.; Liu, Z.; Meng, R.; Chen, X.; Guo, N. Antimicrobial, antioxidant, and antitumor activity of epsi-lon-poly-L-lysine and citral, alone or in combination. Food Nutr. Res. 2016, 60, 31891.
30. Yang, X.; Tian, H.; Ho, C.-T.; Huang, Q. Inhibition of citral degradation by oil-in-water nanoemulsions combined with an-tioxidants. J. Agric. Food Chem. 2011, 59, 6113–6119.

31. Kim, S.H.; Song, H.Y.; Choi, S.J. Influence of structural properties of emulsifiers on citral degradation in model emulsions. Food Sci. Biotechnol. 2019, 28, 701–710.
